# Is The Allergen Really Needed in Allergy Immunotherapy?

**DOI:** 10.1007/s40521-014-0038-5

**Published:** 2014-12-06

**Authors:** Thomas M. Kündig, Ludger Klimek, Philipp Schendzielorz, Wolfgang A. Renner, Gabriela Senti, Martin F. Bachmann

**Affiliations:** 1 Dermatology Department, Zurich University Hospital, Gloriastr. 31, 8091 Zurich, Switzerland; 2 Zentrum für Rhinologie und Allergologie, Wiesbaden, Germany; 3 AREBA Biotech AG, Kilchberg, Switzerland; 4 Center for Clinical Trials, Zürich, Switzerland; 5 National Center for Cancer Care and Research, Hamad Medical Corporation, Doha, Qatar

**Keywords:** Virus-like particles, CpG motifs, Vaccine, Allergy, Allergic rhinitis, Immunotherapy

## Abstract

Immunotherapy for type I allergies is well established and is regarded to be the most efficient treatment option besides allergen avoidance. As of today, different forms of allergen preparations are used in this regard, as well as different routes of application. Virus-like particles (VLPs) represent a potent vaccine platform with proven immunogenicity and clinical efficacy. The addition of toll-like receptor ligands and/or depot-forming adjuvants further enhances activation of innate as well as adaptive immune responses. CpG motifs represent intensively investigated and potent direct stimulators of plasmacytoid dendritic cells and B cells, while T cell responses are enhanced indirectly through increased antigen presentation and cytokine release. This article will focus on the function of VLPs loaded with DNA rich in nonmethylated CG motifs (CpGs) and the clinical experience gained in the treatment of allergic rhinitis, demonstrating clinical efficacy also if administered without allergens. Several published studies have demonstrated a beneficial impact on allergic symptoms by treatment with CpG-loaded VLPs. Subcutaneous injection of VLPs loaded with CpGs was tested with or without the adjuvant alum in the presence or absence of an allergen. The results encourage further investigation of VLPs and CpG motifs in immunotherapy, either as a stand-alone product or as adjuvants for allergen-specific immunotherapy.

## Introduction

Respiratory allergies today are some of the most prevalent chronic diseases [1]. According to the Allergic Rhinitis and its Impact on Asthma (ARIA) Initiative, allergic rhinitis is defined as immunoglobulin (Ig)-E-mediated inflammation of the nasal mucosa with the respective symptoms upon allergen exposure [2]. Apart from allergen avoidance, allergy immunotherapy (AIT) represents the only causative treatment option [3–6].

Allergen-specific immunotherapy over 3–5 years requires numerous allergen applications either subcutaneously or sublingually [7, 8], leading to symptom amelioration, reduced use of medication, and better quality of life [3–6]. Importantly, allergen-specific immunotherapy has long-term effects [9, 10] and is disease modifying, as it can prevent the progression of rhinitis to asthma [11–13]. Despite all of these medical advantages over symptomatic pharmacotherapy, fewer than 5 % of allergy patients choose to get treated with AIT [14–16]. This is mainly because of two disadvantages of current AIT protocols. The first disadvantage is that the numerous injections and visits to a medical office over years are very time consuming for the patient. The second disadvantage is that allergen injection is often associated with adverse reactions, which are typically harmless and not bothersome injection site reactions in subcutaneous immunotherapy (SCIT) and oral or gastrointestinal symptoms in sublingual immunotherapy (SLIT); these are experienced by the majority of patients, but adverse events in rare cases may also range from severe systemic allergic reactions to anaphylaxis [17]. Various novel strategies have been proposed to enhance the efficacy and shorten the course of therapy in order to also enhance its safety. These strategies include the use of recombinant native major allergens, as well as modified hypoallergenic allergens [18], use of allergen-derived peptides representing CD4 T cell epitopes [19, 20, 21••], intralymphatic immunotherapy [22••, 23–26], epicutaneous immunotherapy [27–34, 35••, 36], and also the use of adjuvants more potent than alum, such as monophosphoryl lipid A (MPLA) [37•] or CpG [38••], or the use of allergens displayed on virus-like particles (VLPs) [39•, 40, 41].

All of the above strategies are based on the dogma that AIT requires the allergen. This dogma has been challenged, however, by recent data [41, 42••]. Omitting the allergen from the vaccine would strike two birds with one stone. First, this would make immunotherapy safer by avoiding allergic adverse events. Second, one vaccine could be used for all allergies. The present review will discuss the scientific rationale behind this strategy and the available clinical data.

## Therapy

### Use of VLPs

**Fig. 1:**
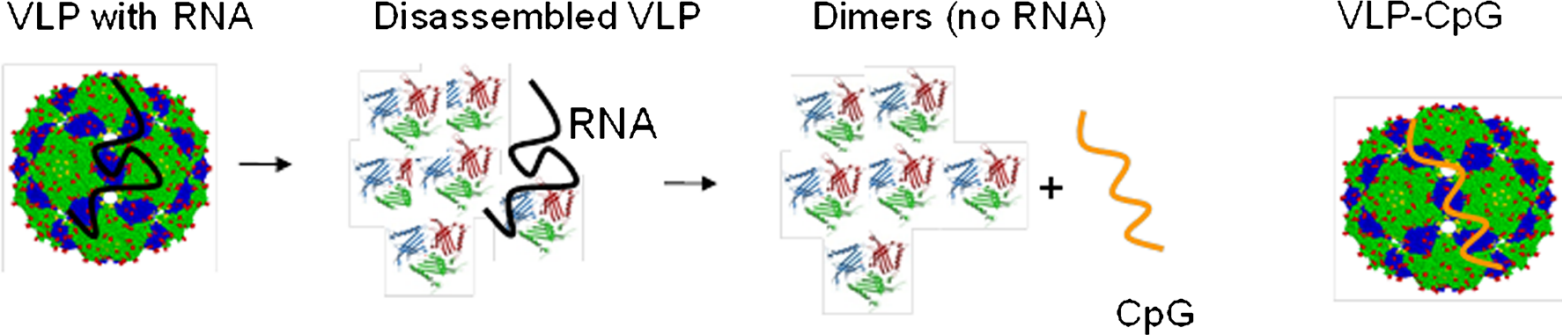
Loading of virus-like particles (VLPs) with CG motifs (CpGs). In a first step, VLPs are disassembled, the RNA is removed in a second step, and the VLPs are finally reassembled in the presence of CpG G10. This results in VLPs loaded with CpGs.

VLPs spontaneously assemble upon expression of virus or bacteriophage capsid proteins (Fig. 1). Some of these VLPs, such as RNA phage-derived VLPs, spontaneously package bacterial RNA upon expression. This RNA may be replaced by DNA rich in nonmethylated CpG motifs (CpGs) [43, 44]. VLPs have proven to be safe and well tolerated and are today broadly used for prophylactic vaccination against hepatitis B virus and human papilloma virus [45••, 46••]. VLPs are also currently being evaluated in therapeutic vaccination against cancer and other noncommunicable chronic diseases, as well as infections [47].

Because of their natural function as carriers of genetic information, virus or bacteriophage capsid-derived VLPs can readily be filled with DNA or RNA. VLPs stabilize and protect these molecules from enzymatic degradation by DNAses and RNAses. Bacteriophage-derived VLPs filled with immunostimulatory CpGs have been used to display tumor antigens and were found to efficiently induce tumor antigen-specific cytotoxic CD8 and CD4 T cells in melanoma patients [48–50]. Similar CpGs were successfully used as adjuvants in prophylactic vaccination to enhance B cell responses [51]. Overall, VLP technology bears great potential for delivery of CpG in cancer, autoimmune disease, and allergies [44]. The present review will focus on clinical experience using bacteriophage VLPs filled with CpGs for treatment of allergic respiratory disease.

### Immune responses induced by VLPs

VLPs can be subcutaneously or intramuscularly injected. Their diameter of approximately 30 nm facilitates entry into lymphatic vessels and direct drainage into local lymph nodes [52–54]. Once in the lymph node, VLPs are taken up by lymph node resident dendritic cells (DCs). Again, this uptake is enhanced by the size and form of VLPs [55]. VLPs are excellent inducers of CD4 T cells via the major histocompatibility complex (MHC) class II pathway, but also cross presentation on the MHC class I pathway is highly efficient [56, 57].

DCs can be subdivided into two main populations. While myeloid DCs (mDCs) produce mainly interleukin (IL)-12, plasmacytoid DCs (pDCs) produce mainly interferon (IFN)-α [58–60]. CpGs are important inducers of cytokines in these cells. There is, however, an important difference between mice and humans. In mice, both pDCs and mDCs express toll-like receptor (TLR)-9, while in humans, only pDCs express TLR9. Hence, IL-12 is hardly induced in humans and the dominant cytokine is usually IFN-α [60, 61]. This has important implications for the type of CpG that may be used for therapy, as outlined below.

Overall, the immune response against VLPs is characterized by immunological properties similar to those seen with live viral vaccines, but, in contrast to the latter, VLPs have the following advantages:

1. The complete lack of genetic information with regard to viral replication enhances safety. In contrast to attenuated vaccines, no reversion to the virulent form is possible.
2. As VLPs assemble spontaneously, they can be produced cheaply in *Escherichia coli* or yeast, in accordance with Good Manufacturing Practice (GMP) guidelines.
3. The size and form of VLPs enhance lymphatic drainage and uptake by antigen-presenting cells in the local lymph nodes.
4. Immunostimulatory CpGs or RNA can be packaged into VLPs and are stabilized and protected from enzymatic digestion inside the VLPs [44]. Many protein and other antigens can readily be displayed on the surface of VLPs. These antigens can either be genetically fused to the VLP protein, or they can be chemically linked to the surface of the VLP. Hence, VLPs may be used as a display platform to render antigens of choice highly immunogenic.

## CpG motifs

The human immune system can be activated by pathogen-associated molecular patterns (PAMPs), which are common to nearly all pathogens. These include nonmethylated CG-rich DNA sequences, which are common in the bacterial genome but not in the genome of vertebrates, where these motifs are usually methylated. Numerous such CpGs have been characterized and can be synthesized as oligodeoxynucleotides (ODNs), using either the naturally occurring “phosphodiester” bonding or the more stable “phosphorothioate” bonding in the backbone of the molecule, hence the designation “C–p–G”. CpGs bind to TLR9, which activates (via the adaptor molecule MyD88) the nuclear factor (NF)-κB pathway, which starts the inflammatory cascade [62].

Three different types of CpGs have been distinguished—namely, A-, B-, and C-type CpGs. Type A CpGs contain their CG motifs in a palindromic sequence, surrounded by guanosines. The backbone of type A CpGs consists of phosphodiester bonds, which are readily digested by DNAses. In contrast, the backbone of type B CpGs consists of phosphorothioate bonds, which are DNAse resistant. Type C CpGs are characterized by phosphorothioate bonds, as well as CG motifs in palindromes on a phosphodiester backbone. These three different types of CpGs differ in their immunological activities. Type A CpGs induce mainly IFN-α, whereas type B CpGs are potent activators of B cells but barely induce IFN-α. In mice, type B CpGs are potent inducers of IL-12 in mDCs. But, as mentioned above, as human mDCs do not express TLR9, the IL-12 response to this type of CpG is inefficient in humans. In addition, pDCs essentially fail to respond to type B CpGs, as they produce only low levels of IL-12 and do not respond with the production of IFN-α to stimulation with this type of CpG. Thus, human DCs respond very poorly to B-type CpGs. Finally, type C CpGs have the characteristics of both A- and B-type CpGs.

## TLR9

While, in mice, TLR9 is expressed in both pDCs and mDCs, in humans, TLR9 is expressed only in pDCs. TLR9 is also expressed in B cells, mast cells, neutrophils, eosinophils, basophils, and keratinocytes [63]. TLR9 is intracellularly located, i.e., within the endosomes, which are accessed by phagocytosed pathogens first. Stimulation of TLR9 polarizes the immune response towards the T-helper (Th)-1 type and stimulates immunoglobulin class switching toward IgG2a in mice [64, 65] and IgG in humans [66]. Th1 cells, as well as IFN-α production, can suppress Th2 cells [67] and downregulate IgE production. TLR stimulation also has direct effects on mast cells, basophils, and eosinophils [68]. The physiological relevance of these interactions is, however, less clear.

## Rationale for use of VLPs as vaccines

The human immune system has evolved to efficiently recognize highly repetitive structures, because such structures are typically found on the surfaces of viruses and bacteria. To speed up the antibody response, such repetitive arrays of epitopes can crosslink such large numbers of B cell surface receptors that antibody production is induced even in the absence of T cell help. Therefore, repetitive antigens, such as those found on the surface of VLPs, are known as “Th-independent antigens” [69••]. In addition, antigen-presenting cells have evolved to efficiently take up particles of viral size and VLP content is scanned by TLRs in the endosomes. The innate humoral immune system has finally also evolved to recognize repetitive structures, enhancing both B cell activation and uptake/activation of antigen-presenting cells [44].

## Clinical trials with CpGs in immunotherapy for allergic rhinitis

### Allergen covalently coupled to CpGs

Creticos et al. performed a clinical trial using the major ragweed allergen Amb a 1 conjugated to a type B CpG-ODN in 25 ragweed-allergic patients [38••]. In this randomized, double-blind, placebo-controlled phase II study, adult patients received a total of six injections at weekly time intervals. The primary endpoint, which was vascular permeability as measured by the serum albumin concentration in nasal lavage, was not significantly ameliorated in this trial. However, clinical symptoms of allergic rhinitis were ameliorated and quality-of-life scores improved already during the first season after treatment. This improvement persisted also in the following pollen season, i.e., 2 years after vaccination. The treatment proved safe and well tolerated, with no severe adverse events. The authors also observed absence of the IgE boost usually observed after the ragweed season in the treatment group. The highly encouraging phase II clinical data could, however, not be reproduced in a larger phase III clinical trial [70]. This may have been due to the fact that the ragweed pollen counts were particularly low in the relevant season and symptoms consequently were very minor also on placebo. On the other hand, use of B-type CpGs, which inefficiently stimulate human DCs, may have resulted in reduced clinical efficacy (Table 1).

**Table 1:** Clinical trials using virus-like particles (VLPs) and CG motifs (CpGs) [41]

Vaccine	Treatment arms	Patients and disease	Study design	Reference
Qβ VLP coupled to Der p 1-derived peptide (Qβ-Der p 1). Note that this VLP was loaded with *E. coli* RNA	i.m. 50 μg Qβ-Der p 1 (*n* = 6), s.c. 50 μg Qβ-Der p 1 (*n* = 6), i.m. 10 μg Qβ-Der p 1 (*n* = 6), s.c. 10 μg Qβ-Der p 1 (*n* = 6)	24 volunteers; healthy	Randomized, monocentric, open-label	[39•]
Amb a 1 conjugated to type B CpG-ODN	Verum (*n* = 14), placebo (*n* = 11)	25 patients; allergic to ragweed; rhinitis	Randomized, double-blind, placebo-controlled	[38••]
Qβ VLP filled with type A CpG-ODN (QbG10) + HDM extract	QbG10 + HDM allergen (*n* = 20)	20 patients; allergic to HDM; rhinoconjunctivitis	Randomized, monocentric, open-label	[71]
Qβ VLP filled with type A CpG-ODN (QbG10)	0.5 mg QbG10 (*n* = 99), 1 mg QbG10 (*n* = 103), placebo (*n* = 97)	299 patients; allergic to HDM; rhinoconjunctivitis	Randomized, double-blind, placebo-controlled	[42••]

*HDM* house dust mite, *i.m.* intramuscular, *ODN* oligodeoxynucleotide, *s.c.* subcutaneous

### Allergen conjugated to VLPs filled with RNA

We have performed an open-label phase I clinical trial with a house dust mite (HDM) Der p 1-derived peptide of 16 amino acids in length, which was covalently coupled by a chemical linker to bacteriophage Qβ-derived VLPs filled with *E. coli*-derived RNA ligand for TLR7 and 8. The vaccine was administered three times at 4-week time intervals. Twenty-four healthy volunteers participated in this study, and antibody titers were followed for 18 months [39•]. The vaccine was well tolerated, and we observed only local reactions at the site of injection. Already 4 weeks after the first injection, we observed a rapid Der p 1-specific serum IgM and IgG response. Interestingly, the IgG subclasses were mainly IgG1 und IgG3—thus those subclasses typically observed after viral infection, rather than the IgG4 response typically observed after conventional AIT. We observed no differences between the intramuscular and the subcutaneous routes of injection [39•] (Table 1). It would be interesting to repeat this study with VLPs loaded with CpGs rather than RNA.

### Allergen admixed to VLPs filled with CpGs

In an additional clinical trial, we investigated the safety and tolerability, as well as the clinical efficacy, of type-A CpGs packed in Qβ-derived VLPs. The CpG-loaded VLPs were mixed with HDM allergens and used as an adjuvant in specific immunotherapy [71] (Table 1). In order to keep the formulation simple, CpG-containing VLPs were simply mixed with a conventional HDM extract. Twenty HDM-allergic patients were included in this open-label, monocentric clinical trial. The first few injections were performed with the HDM extract only, following the conventional cluster regimen. This short uptitration phase was followed by a total of six weekly injections of HDM extract mixed with VLPs containing CpGs. The clinical endpoints of this trial were conjunctival provocation testing, as well as monitoring of allergic rhinitis and asthma, quality-of-life questionnaires, skin prick tests and antibody measurements. We found a good safety and tolerability profile. Symptoms of allergic rhinitis and asthma were significantly reduced, and allergen-specific IgG was found to increase. Allergen-specific IgE, after an initial rise, was subsequently also found to decrease [71]. We repeated that clinical trial in a double-blind and placebo-controlled manner. In this clinical trial, HDM extract mixed with CpG-containing VLPs was compared with HDM extract alone or with CpG-containing VLPs alone, or with another arm containing only placebo (Senti et al., unpublished). This trial could essentially confirm the results of the open-label study. We were surprised, however, to find that the clinical effects with VLPs filled with CpG alone were as good as those obtained when the HDM extract was admixed. These puzzling results were the starting point of a clinical trial program where the allergen was to be omitted from allergen-specific immunotherapy.

In the following clinical trial, we therefore used the same Qβ-derived VLPs filled with the same type A CpG, named G10. The study was a randomized, double-blind, placebo-controlled phase IIb trial in 299 patients suffering from allergic rhinoconjunctivitis due to HDM allergy [42••] (Table 1). Patients received 6-weekly injections either with verum or placebo. The study duration per patient was 9 weeks. The clinical endpoints were the symptom and medication scores and quality-of-life scores, as well as conjunctival provocation testing. The treatment proved to be safe and well tolerated. Symptom and medication scores improved significantly in the high-dose verum group, confirming that treatment with VLPs filled with CpGs alone could ameliorate allergic rhinitis, even without injection of allergen.

## Possible mechanisms of immunotherapy without allergens

Animal models suggest that type-A CpG-induced Th1 responses and IFN-α inhibit Th2 responses. Furthermore, symptom amelioration could also be due to direct effects of CpGs on mast cells, which also express TLR9 [72–74]. CpGs have also been found to change the enzymatic activity of indoleamine 2,3 dioxygenase (IDO), an enzyme known to play an important role in T cell regulation [75•]. Following CpG-induced IFN-α production, the cytokine was found to upregulate IDO in DCs, leading to T cell suppression [76]. This mechanism may explain some of the inflammation-inhibiting effects of CpGs in allergic diseases. On an epidemiological level, the “hygiene hypothesis” supports the notion that bacterial byproducts have inhibitory effects on the development of allergies, at least early in life [77••]. It is well known that children born and raised on farms have a significantly lower risk of respiratory allergies and asthma. The same is true for children born into households with many children, as well as households with pets, and for children visiting day care centers [78–80]. The persistent protective effect of exposure to bacterial byproducts early in life may be explained by epigenetic modifications [81] or by long-lived Th1 cells, creating a cytokine milieu disfavoring the development of Th2 cells. CpGs have also been found to activate ICOS ligands on pDCs, which represents an important costimulatory molecule for the induction of regulatory T cells [82].

Last ,but not least, it should not be forgotten that if clinical trials are being performed in HDM allergy, the latter allergens are nearly ubiquitous and present during the entire season. It can therefore not be excluded that the natural exposure to allergen plays a role in the “allergen-free” treatment regimens being discussed.

Thus, treating allergies with CpG-loaded VLPs remains an attractive option and may allow desensitization of a large number of patients in an efficient manner in the absence of risk of anaphylactic reactions. However, there are a number of questions that still need to be resolved, including finding the optimal dose and the duration of the therapeutic effect. Further clinical trials are therefore required to elucidate whether “allergen-free” immunotherapy is a valuable long-term treatment option for allergies.
